# The impact of lowering the cut-off value on the sensitivity of the Platelia Elisa IgG (Bio-Rad) test for toxoplasmosis diagnosis

**DOI:** 10.1051/parasite/2015022

**Published:** 2015-07-17

**Authors:** Oussama Mouri, Eric Kendjo, Feriel Touafek, Arnaud Fekkar, Ousmane Konte, Sebastien Imbert, Régis Courtin, Dominique Mazier, Luc Paris

**Affiliations:** 1 AP-HP, Groupe Hospitalier La Pitié-Salpêtrière, Service de Parasitologie-Mycologie 75013 Paris France; 2 Centre d’Immunologie et des Maladies Infectieuses, CIMI-Paris 75013 Paris France; 3 Sorbonne Universités, UPMC Université Paris 6, CIMI-Paris 75005 Paris France

**Keywords:** Toxoplasmosis, *Toxoplasma gondii*, Congenital toxoplasmosis, Pregnant women, Western blot

## Abstract

Determining specific immune status against *Toxoplasma gondii* is essential for assessing the risk of reactivation in immunocompromised patients or defining serological monitoring and appropriate prophylactic measures during pregnancy. In France, toxoplasmosis serological screening requires systematic testing for IgM and IgG antibodies. The Platelia Toxo IgG and IgM test (Bio-Rad) is one of the most widely used tests for anti-toxoplasmic antibody detection. We performed a study on 384 sera, including 123 IgG negative (<6 IU/mL) and 261 IgG equivocal (6–9 IU/mL) sera tested with Platelia Toxo IgG and collected during routine screening at Pitié-Salpêtrière Hospital, Paris, France to determine the best-performing IgG titer cut-off value. Out of these 383 sera, 298 were IgM negative by Platelia Toxo IgM and 86 were IgM positive. All sera were also tested against Toxo IgG II LD BIO western blot test as confirmation. Our results indicated that an IgG titer cut-off value of ≥4.4 IU/mL for the Platelia Toxo IgG met the definition of positivity, a value significantly lower than that indicated by the manufacturers. In the presence of IgM antibodies, the IgG titer cut-off decreased significantly to a value ≥0.2 IU/mL. This latter cut-off also allowed adequate diagnosis of proven toxoplasmosis seroconversion in 76.7% of cases (33/43). Our findings may improve toxoplasmosis care by reducing therapeutic intervention time and eliminating the need for further serological monitoring.

## Abstract (fr)

La détermination du statut immunitaire spécifique contre *Toxoplasma gondii* est essentielle pour évaluer le risque de réactivation chez les patients immunodéprimés ou définir la surveillance sérologique et les mesures prophylactiques appropriées pendant la grossesse. En France, le dépistage sérologique de la toxoplasmose repose sur la mise en évidence des IgG et IgM antitoxoplasmiques. La technique Platelia Toxo IgG (Bio-Rad) est l’un des tests les plus couramment utilisés pour la détection des anticorps anti-toxoplasmose. Nous avons réalisé une étude sur 384 sérums dont 123 étaient IgG négatifs (< 6 UI/mL) et 261 étaient douteux (6–9 UI/mL) avec la technique Platelia Toxo IgG, collectés dans le cadre de l’activité de routine des Hôpitaux Pitié Salpêtrière à Paris, France, afin de déterminer la valeur la plus discriminante du seuil de positivité pour le titre des IgG. Parmi les 384 sérums, 298 étaient IgM négatifs et 86 étaient IgM positifs avec la technique Platelia Toxo IgM. Tous les sérums ont été systématiquement testés en Western Blot avec la trousse LD BIO Toxo IgG II LDBIO utilisée comme test de référence. Nos résultats montrent qu’un titre d’IgG ≥ 4.4 UI/mL pour le Platelia Toxo IgG définit le seuil de positivité de la technique, une valeur nettement inférieure à celle recommandé par le fabricant (9 UI/mL). En présence d’IgM, le seuil de positivité du titre des IgG baisse de manière significative à ≥ 0.2 UI/mL. Ce dernier seuil a permis le diagnostic de 76.7 % (33/43) des séroconversions toxoplasmiques avérées. Nos résultats peuvent améliorer le soin de la toxoplasmose en réduisant la durée des interventions thérapeutiques et en éliminant le besoin de surveillance sérologique supplémentaire.

## Introduction

The intracellular parasite *Toxoplasma gondii* is the causal agent of toxoplasmosis, the most widespread protozoan infection in humans. For most populations, toxoplasmosis is usually benign. However, acquired infections are potentially serious and often lead to severe complications both in non-immune pregnant women, where they may lead to congenital toxoplasmosis, and in people with compromised immune systems, particularly patients with non-controlled HIV infection, hematopoietic stem cell transplantation, or solid organ transplantation. Furthermore, reactivation from previous infections is also common during immunodeficiency. Toxoplasmosis is usually diagnosed via the detection of IgG and IgM anti-*Toxoplasma* antibodies. Their presence is used to assess the risk of toxoplasmosis reactivation (immunocompromised patients) or to determine immunity during pregnancy, and their absence is used to define serological monitoring and appropriate prophylactic measures. Immunodiagnostic assays are valuable, widely used tools, but the results obtained across the different IgG assays, reported as IU/mL, may differ markedly because of a lack of standardization. During acute infection, high or rising IgG antibody titers aid in the diagnosis whereas very low titers, considered equivocal, do not permit differentiation between old infections and an absence of immunization [2, 9, 11].

The Platelia Toxo IgG (Bio-Rad) test is one of the most widely used tests for anti-toxoplasmic antibody detection [3]. However, previous studies performed in our laboratory or reported by others authors have recently suggested that the cut-off for this test as defined by the manufacturer is too high, leading to unsatisfactory sensitivity [8, 10]. We thus performed a large study on 384 sera to determine the best-performing cut-off values for this test.

## Materials and methods

The study took place between January and December 2011 in the Parasitology-Mycology laboratory of the Pitié-Salpêtrière Hospital, a 1800-bed tertiary care medical center in Paris, France. During this period, 384 IgG negative (<6 IU/mL) or equivocal (6–9 IU/mL) serum samples were tested with Platelia Toxo IgG.

The sera were collected as a part of routine laboratory tests from: (1) pregnant women during routine screening and their newborns during a follow-up (*n* = 214); (2) immunocompromised patients (*n* = 48); (3) samples taken from patients at other laboratories referred to our institution within its role as network member of the national reference center for toxoplasmosis for the determination of their serological status (*n* = 122).

In France, toxoplasma serological screening requires systematic testing for IgM antibodies in addition to IgG (*national health authority recommendations*). Out of these 384 sera, 298 were IgM negative (Group I) and 86 were IgM positive (Group II).

The sera were tested systematically with Platelia Toxo IgG and IgM (Bio-Rad) on an automated analyzer (Etimax, Diasorin). The LD BIO Toxo IgG II (LDBIO Diagnostics) western blot was used as a reference confirmatory test for the determination of anti-*Toxoplasma* antibodies.

### Laboratory tests

#### Immunoblot

The LDBio Toxo IgG II test is a qualitative test in which parasite antigens are separated by electrophoresis and then transferred by electroblotting to nitrocellulose strips. The resulting bands on the strips of patients are compared to the five bands on a positive control strip, representing 30, 31, 33, 40, and 45 kDa. A positive result following the manufacturer’s recommendations was defined by the presence of at least three matching bands on the patient strip, including necessarily the band at 30 kDa.

### Immunodiagnostic assay

#### Platelia Toxo IgG

The Platelia Toxo IgG is a solid-phase enzyme-linked immunosorbent assay (ELISA) for the detection and titration of anti-*Toxoplasma* IgG. The antigenic composition is a mixture of antigens and the secondary antibody is a specific class peroxidase-conjugated anti-human IgG. The results are expressed in international units per milliliter (IU/mL). In the present study, titers >6 and <9 IU/mL were considered equivocal and those ≥9 IU/mL were considered positive, as per the manufacturer’s instructions.

#### Platelia Toxo IgM

The Platelia Toxo IgM is a qualitative test for the detection of IgM antibodies against *T. gondii* via the capture of IgM in the solid phase (the microplate wells are coated with anti-human *μ* chains). A mixture of antigens and the monoclonal anti-*T. gondii* antigen antibody labeled with peroxidase is used as the conjugate. In the present study, ranges >0.8 to <1 were considered equivocal and those ≥1 were considered positive, as per the manufacturer’s instructions.

### Statistical analysis

The discriminative ability of the immunoenzymatic test for diagnostic accuracy of toxoplasmosis (presence of anti-*Toxoplasma* antibodies) in each serum was assessed by means of the receiver operating characteristic (ROC) curve [4, 6] and the area under the curve (AUROC) was calculated as a measure of the validity of threshold positivity for the Platelia Toxo IgG Kit.

As a first approach, cut-off values of IgG titers were obtained by means of the evaluation of the likelihood ratio (LR) (ratio between sensitivity and specificity). The highest LR values were used as cut-offs in the studied sera for toxoplasmic serology (positive). These results were then stratified into the three groups (pregnant women, immunocompromised, and other patients) to confirm our findings.

Pearson’s chi-square test was used to test the association between the 0.2 IU Elisa IgG cut-off and seroconversion.

Stata version 12 (StataCorp, College station, TX, USA) was used for all statistical analyses. All reported *p*-values are two sided. Only *p*-values <0.05 were considered statistically significant.

## Results

### General description

Overall, 384 sera were collected, including 123 IgG negative (<6 IU/mL) and 261 IgG equivocal sera (6–9 IU/mL) tested with Platelia Toxo IgG, according to the manufacturer’s instructions. Out of these, 262 (68.2%) were found to be positive and 122 (31.8%) were negative (no band or insufficient number) with the Toxo II IgG Western blot.

#### IgG negative serum samples

Of the 123 IgG negative sera samples tested with Platelia Toxo IgG, according to the manufacturer’s instructions, 18 (14.63%) were found to be positive and 105 (85.37%) were negative (no band or insufficient number) with the Toxo II IgG western blot.

#### IgG equivocal serum samples

Of the 261 IgG equivocal (6–9 IU/mL) serum samples tested with Platelia Toxo IgG, according to the manufacturer’s instructions, 244 (93.49%) were found to be positive and 17 (6.51%) were negative (no band or insufficient number) with the Toxo II IgG western blot.

#### Analysis of IgG titer cut-off value

**Figure 1.**
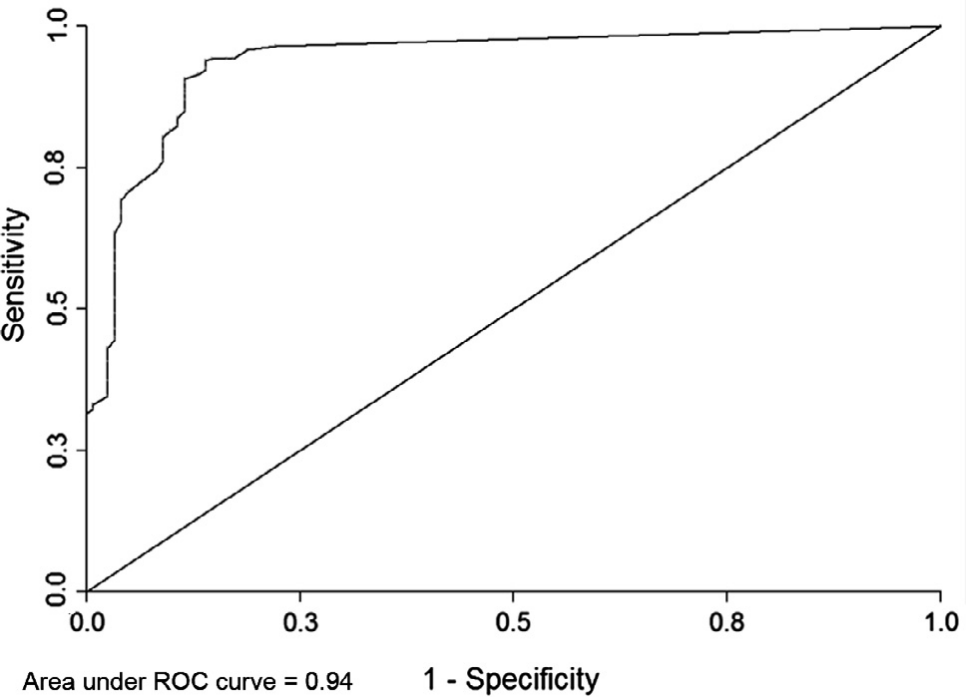
Receiver operating characteristic curve for 384 IgG negative (<6 UI/mL) or equivocal sera (6–9 UI/mL).

The overall analysis showed that a lower IgG titer cut-off of 4.4 IU/mL (LR = 40.68) was able to classify an additional 30.7% (86/260) of sera samples as positive, which were previously identified as equivocal or negative with the manufacturer-suggested cut-offs of Platelia Toxo IgG, but positive with the western blot. Sensitivity was 33.1%, specificity was 99.2%, and positive predictive value (PPV) was 99.0% for the Platelia Toxo IgG immunoassays using the 4.4 IU/mL cut-off (Fig. 1).

### Effect of the presence or the absence of IgM antibody on the IgG titer cut-off

**Figure 2.**
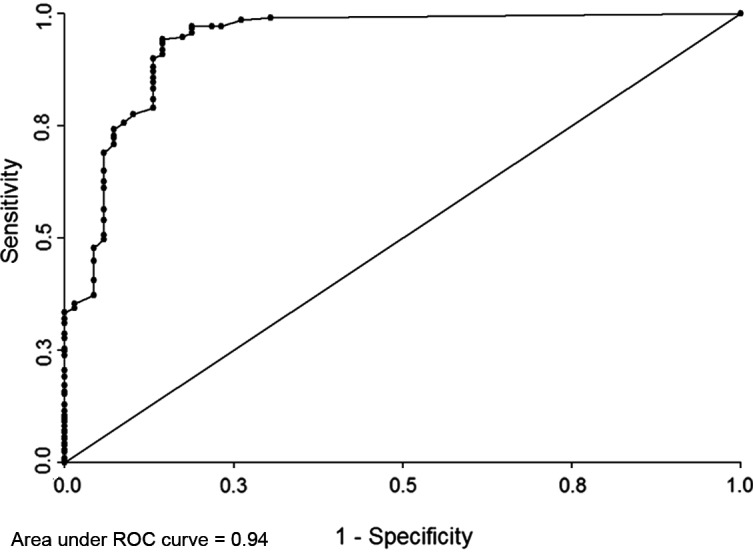
Receiver operating characteristic curve for 298 IgG negative (<6 UI/mL) or equivocal (6–9 UI/mL) and IgM negative sera.

The analysis of Group I showed that there was no significant variation in the IgG titer cut-off, which remained at 4.4 IU/mL (LR+ of 30.1). This cut-off was able to classify 35.4% (75/212) of the sera previously considered equivocal or negative with the manufacturer-suggested cut-offs of Platelia Toxo IgG, but as positive with the western blot. Sensitivity was 35.4%, specificity was 98.8%, and PPV 98.7% for the Platelia Toxo IgG immunoassays using the 4.4 IU/mL cut-off (Fig. 2).

**Figure 3.**
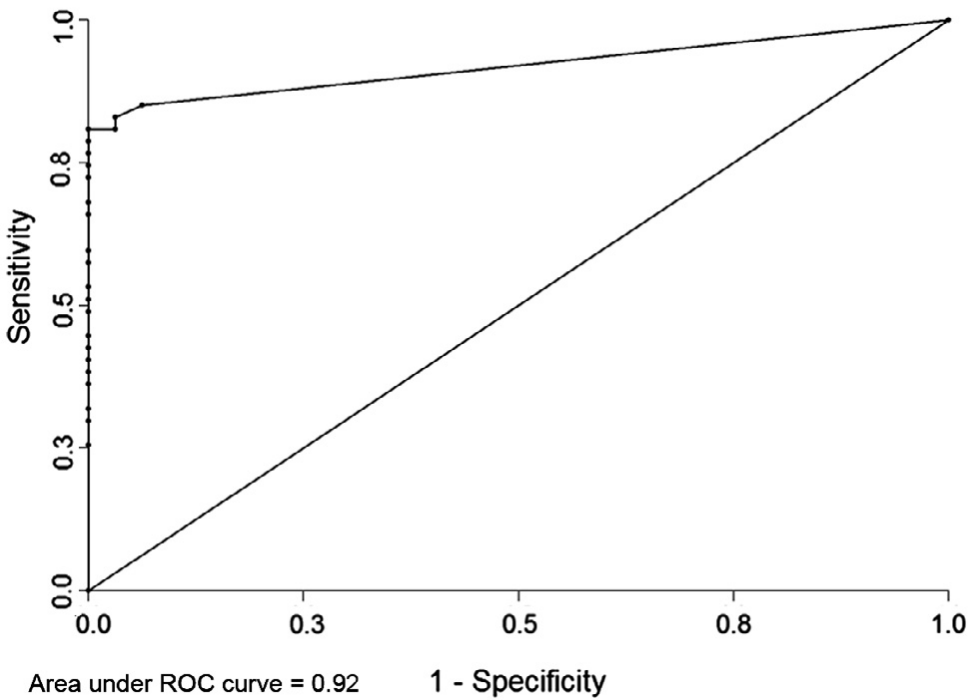
Receiver operating characteristic curve for 86 IgG negative (<6 UI/mL) or equivocal (6–9 UI/mL) and IgM positive sera.

In Group II however, there was a significant change in the IgG titer cut-off, which decreased from 4.4 to 0.2 IU/mL (LR+ of 31.7). This cut-off value was able to classify 83.3% (40/48) of the sera previously considered equivocal or negative with the manufacturer-suggested cut-offs of Platelia Toxo IgG, but as positive with the western blot, with an increase in sensitivity (83.3%). Specificity was 97.4% and PPV 97.4% for the Platelia immunoassays using the 0.2 IU/mL cut-off (Fig. 3).

### Association between the 0.2 IU/mL IgG titer cut-off and toxoplasmosis seroconversion

**Figure 4.**
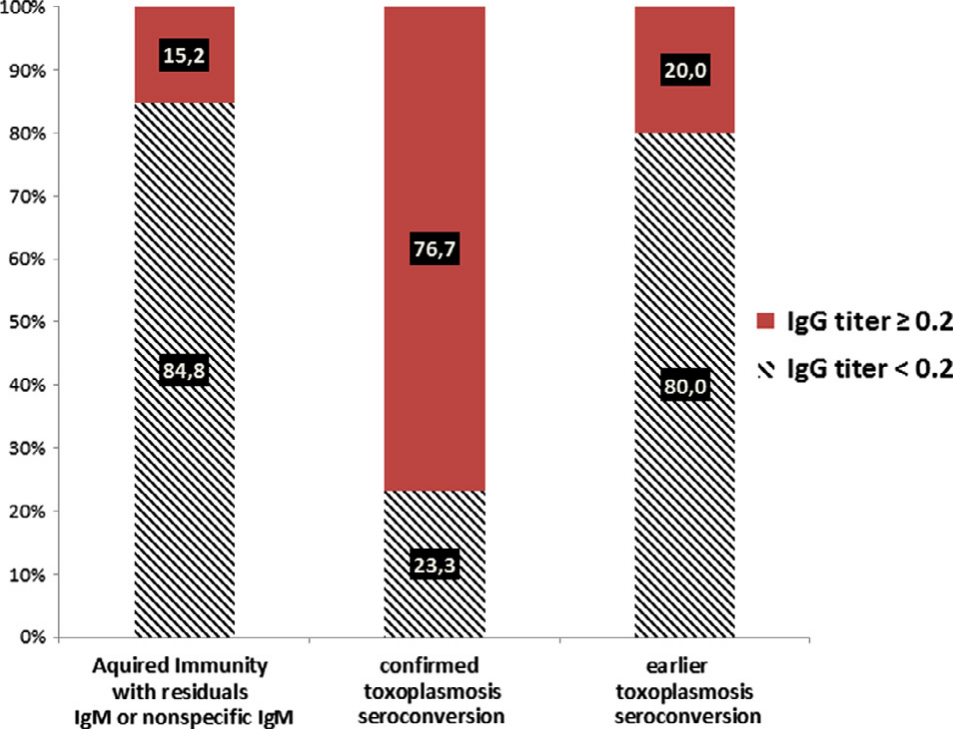
Distribution of toxoplasmosis seroconversion by IgG titer cut-off ≥0.2 IU/mL for 86 IgG negative (<6 UI/mL) or equivocal (6–9 UI/mL) and IgM positive sera.

The analysis in Group II showed that an IgG titer cut-off of 0.2 IU/mL was significantly associated with toxoplasmosis seroconversion (*P* < 0.001) for patients with a previous negative sample. This cut-off is able to correctly diagnose proven toxoplasmosis seroconversion in 76.7% (33/43) of cases and earlier seroconversion in 20% (2/10) of cases (Fig. 4).

### Analysis of IgG titer cut-off value within clinical groups

#### I. Pregnant women

Analysis of the pregnant women group showed that a lower IgG titer cut-off of 4.4 IU/mL was able to classify 52% of sera samples as positive. Sensitivity was 32%, specificity 98%.

##### Effect of the presence or the absence of IgM on the IgG titer cut-off

In the absence of IgM (150 sera), there was no significant variation in the IgG titer cut-off, which was 4.4 IU/mL. This cut-off was able to classify 52% of sera samples as positive. Sensitivity was 31%, specificity 97%.

In the presence of IgM (64 sera), a lower IgG titer cut-off of 0.2 IU/mL was able to classify 87.50% of sera samples as positive. Sensitivity was 79%, specificity 100%.

#### II. Immunocompromised patients

Analysis of the immunocompromised patient group showed that a lower IgG titer cut-off of 4.5 IU/mL was able to classify 58% of sera samples as positive, with a sensitivity of 42% and specificity of 100%.

##### Effect of the presence or the absence of IgM on the IgG titer cut-off

In the absence of IgM (39 sera), there was no significant variation in the IgG titer cut-off; its value was 4.5 IU/mL. This cut-off was able to classify 48% of sera samples as positive. Sensitivity was 41%, specificity 100%.

#### III. Other clinical situations

Analysis of this group showed that a lower IgG titer cut-off of 4.5 IU/mL was able to classify 56% of sera samples as positive. Sensitivity was 29%, specificity 100%.

##### Effect of the presence or the absence of IgM on the IgG titer cut-off

In the absence of IgM (101 sera), there was no significant variation in the IgG titer cut-off, which remained at 4.3 IU/mL. This cut-off was able to classify 59% of sera samples as positive. Sensitivity was 29%, specificity 100%.

In the presence of IgM (21 sera), a lower IgG titer cut-off of 0.2 IU/mL was able to classify 85% of sera samples as positive. Sensitivity and specificity were, respectively, 90% and 81%.

## Discussion

Determining specific immune status is essential for evaluating and preventing the risk of severe toxoplasmosis complications in susceptible individuals such as non-immune pregnant women or immunocompromised patients. Immunoenzymatic tests are the most frequently used serological diagnostic toxoplasmosis methods. However, commercial reagents vary considerably in detecting low concentrations of antibodies. The Toxo II IgG test was developed by LDBio to confirm serological results for low titers of IgG. A previous study evaluated its use as confirmatory test [2, 8].

The findings of the present study indicate that, in the absence of IgM, an IgG titer cut-off value of ≥4.4 IU/mL with the Platelia Toxo IgG kit – a value significantly less than that indicated by the manufacturer – met the definition of positivity. The PPV was 99.0%, essentially eliminating the risk of false positives. In this situation, it is important to use a reference test, i.e., Toxo II IgG western blot or a Sabin-Feldman test to give a clear conclusion about the immunological status against Toxoplasmosis. This is critical for ensuring proper management of seronegative pregnancies: first, in the implementation of primary prophylaxis and in monthly monitoring to prevent toxoplasmosis infection, and second, in providing proper follow-up, should infection occur.

Our results suggest that the lower threshold of Platelia Toxo IgG should be reduced from 9 to 4.4 IU/mL, and thus confirm the results of Leslé et al. and more recently those reported by Khammari et al. [8, 10]. In the absence of IgM, an IgG titer cut-off value of ≥4.4 IU was able to classify 35.4% (75/212) of the sera previously considered equivocal or negative with the manufacturer-suggested cut-offs of Platelia Toxo IgG, but as positive with the western blot, essentially eliminating the risk of false negative. Therefore, they may also reduce healthcare expenses by eliminating the need for further serological monitoring in patients who are considered to be immunized.

The presence of IgM combined with a very low IgG titer is highly suggestive of early seroconversion for toxoplasmosis, which is confirmed if there is a prior negative sample; toxoplasmosis seroconversion was defined as the emergence of specific immunoglobulin IgG in previously seronegative women or as a significant rise in IgG in women with specific IgM [7]. The simultaneous presence of low IgG and IgM titers caused a significant variation in the IgG titer cut-off, which decreased to a value of 0.2 IU/mL. This latter cut-off also allowed adequate diagnosis of proven toxoplasmosis seroconversion in 76.7% of cases (33/43).

The subgroup analysis showed that clinical status of the patient did not significantly modify the value of the cut-off. This result was consistent with the overall analysis.

In 1978, the French health authorities implemented a prevention program for congenital toxoplasmosis (CT), recommending systematic serological testing during the first trimester of pregnancy. Since 1992, the recommendations have included monthly serological screening of seronegative pregnant women from diagnosis of pregnancy until delivery [1].

Historically, the prevention of parasite transmission to the fetus was the aim of prenatal screening. However, a recent study found that early treatment of primary infection during pregnancy had little or no effect on the fetomaternal transmission rate, but did reduce the incidence of sequelae in infected infants. Some authors have hypothesized that the ineffectiveness of prenatal treatment may be due to transmission before the start of treatment [5, 7]. Our study showed that in the presence of IgM, an IgG titer cut-off value of ≥0.2 IU/mL met the definition of positivity if the patient *was known previously seronegative for toxoplasmosis with IgG titer equal to 0 IU/mL*; this cut-off was *highly* suggestive of early or proven toxoplasmosis seroconversion and able to confirm seroconversion in infected mothers several weeks earlier.

However, in this situation, it is important to use an additional confirmatory test, i.e., Toxo II IgG western blot or a Sabin-Feldman dye test and serological control 10–15 days later to support this hypothesis. Such an approach would allow earlier diagnosis of toxoplasmosis seroconversion and thus earlier treatment (less than four weeks), which can be particularly important in pregnant women to reduce disease-related effects in children. In the absence of IgM, an IgG titer cut-off value of ≥4.4 IU may also reduce healthcare costs by eliminating the need for further serological monitoring [2, 7, 10].

## Conflict of interest

The authors have no relationships (commercial or otherwise) that might constitute dual or conflicting interests for the present study.
